# *Bartonella* infection in small mammals and their ectoparasites from the Central Highlands of Madagascar: diversity and implication in future zoonotic surveillance

**DOI:** 10.1186/s13071-025-07233-9

**Published:** 2026-02-06

**Authors:** Lanto Andrianarijaona Maminirina, Mamionah N. J. Parany, Fanohinjanaharinirina Rasoamalala, Angelo Andrianiaina, Lalatiana O. Randriamiharisoa, Mercia Rasoanoro, Soloandry Rahajandraibe, Voahangy Soarimalala, Milijaona Randrianarivelojosia, Dina Ratahiriarisoa, Minoarisoa Rajerison, Steven M. Goodman, Beza Ramasindrazana

**Affiliations:** 1 Institut Pasteur de Madagascar, Antananarivo, Madagascar; 2 Faculté des Sciences, Université de Fianarantsoa, Fianarantsoa, Madagascar; 3 Université d’Antananarivo, Antananarivo, Madagascar; 4 Madagascar National Parks, Antananarivo, Madagascar; 5 Association Vahatra, Antananarivo, Madagascar; 6 Ministère de la Santé Publique, Antananarivo, Madagascar; 7 Université de Toliara, Toliara, Madagascar; 8 Field Museum of Natural History, Chicago, IL USA

**Keywords:** *Bartonella*, Fandriana and Ankazobe districts, Fleas, Small mammals, Zoonoses

## Abstract

**Background:**

This research aimed to investigate the prevalence and diversity of *Bartonella* in small mammals and their ectoparasites from the Central Highlands of Madagascar and to refine existing information on potential associated zoonotic diseases.

**Methods:**

A retrospective analysis was performed on mammals and their ectoparasites collected in the Fandriana and Ankazobe districts, including 253 spleen samples from seven small mammal species and 183 individual ectoparasites (132 fleas and 51 ticks). Genomic DNA was extracted and amplified by polymerase chain reaction (PCR) targeting the *nuoG* gene (346 bp). Sanger sequencing of the PCR products was performed to assess *Bartonella* diversity using phylogenetic analysis.

**Results:**

In total, 60.1% (152/253) of small mammals and 15.9% (21/132) of fleas tested positive for *Bartonella*, with *Rattus rattus* (69.1%, 137/198) and the associated flea *Synopsyllus fonquerniei* (21.2%, 14/66) having the highest infection rates. At the same sampled locations, adult *R. rattus* were more frequently infected with *Bartonella* than juveniles. Phylogenetic analysis revealed five associated clades of *Bartonella* with two clades recognized as a potential zoonotic species (*B. elizabethae* and *B. kosoyi*).

**Conclusions:**

Using molecular tools, we report a high prevalence of *Bartonella* in small mammals and their fleas in the Central Highlands of Madagascar. Two potential *Bartonella* zoonotic species were identified in *R. rattus* and their fleas. As these bacteria are generally vector-borne, they could have a significant impact on public health in the vicinity of our study areas and, in general, in Madagascar, and merit further investigation.

**Graphical Abstract:**

**Figure:**
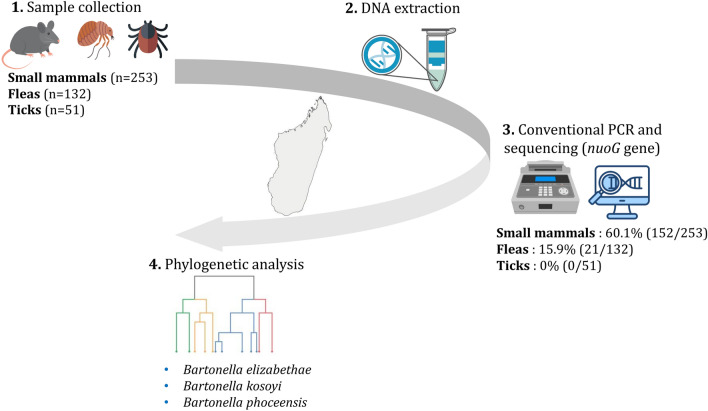

**Supplementary Information:**

The online version contains supplementary material available at 10.1186/s13071-025-07233-9.

## Background

Small mammals are recognized as reservoirs of different zoonotic bacteria that cause infectious diseases in humans, such as leptospirosis (*Leptospira* spp.), plague (*Yersinia pestis*), murine typhus (*Rickettsia typhi*), Lyme disease (*Borrelia* spp.), and bartonellosis (*Bartonella* spp.) [1]. Members of the genus *Bartonella* (family Bartonellaceae; order Rhizobiales) are emerging vector-borne zoonotic bacteria of medical and veterinary importance that are distributed worldwide and represent a global public health concern [2, 3]. These microorganisms are fastidious, Gram-negative, facultative intracellular bacteria with a marked tropism for erythrocytes and endothelial cells [4, 5]. They infect a broad range of vertebrate hosts, with small mammals, particularly rodents, shrews, and bats, acting as the principal reservoirs [4, 6]. Infections have also been reported in other mammals, including carnivorans and humans [7]. Small mammals, especially rodents, harbor the greatest diversity of *Bartonella* species, facilitating transmission within the mammalian community or to humans upon exposure [2, 8]. Globally, approximately 90 rodent species are recognized as hosts for more than 22 species of *Bartonella* [4, 9]. Currently, the List of Prokaryotic names with Standing in Nomenclature (LPSN) records 42 species and subspecies of *Bartonella* [10], of which at least 15 are associated with human diseases [7]. Transmission to humans and other animals occurs through blood-sucking arthropod vectors (such as fleas, ticks, lice, and sandflies), direct contact with infectious feces of arthropod vectors, or scratches or bites of infected animals [2, 5].

Among the described species of *Bartonella*, *B. rattimassiliensis*, *B. rochalimae*, *B. elizabethae*, *B. grahamii*, *B. tamiae*, *B. vinsonii* subsp. *arupensis*, *B. vinsonii* subsp. *berkhoffii*, *B. birtlessi*, *B. tribocorum*, and *B. washoensis* are associated with rodents and may cause fever, endocarditis, lymphadenitis, neuroretinitis, myocarditis, and meningitis in humans [2, 4, 7, 8].

Madagascar is a biodiversity hotspot and accordingly also for zoonotic risks owing to important levels of human–wildlife contact, environmental change, and the presence of multiple potential reservoirs and vectors [11–17]. Despite the increasing worldwide recognition of *Bartonella* associated with emerging zoonotic agents, available data from Madagascar are notably limited. A few studies have reported the presence of *Bartonella* in fruit bats [18], rodents [19], and mammal ectoparasites [20]. However, the prevalence, diversity, and potential risk to public health associated with *Bartonella* in small mammals and their ectoparasites in Madagascar are still poorly understood.

Understanding the circulation of *Bartonella* in small mammals and their ectoparasites is crucial to assess their diversity, elucidate their role in zoonotic transmission, anticipate potential outbreaks, and provide insights into their impact on public health. Herein, we investigated the prevalence and diversity of *Bartonella* in small mammals and their ectoparasites to provide new insights into their potential implication for public health in Madagascar.

## Methods

### Study sites

**Fig. 1:**
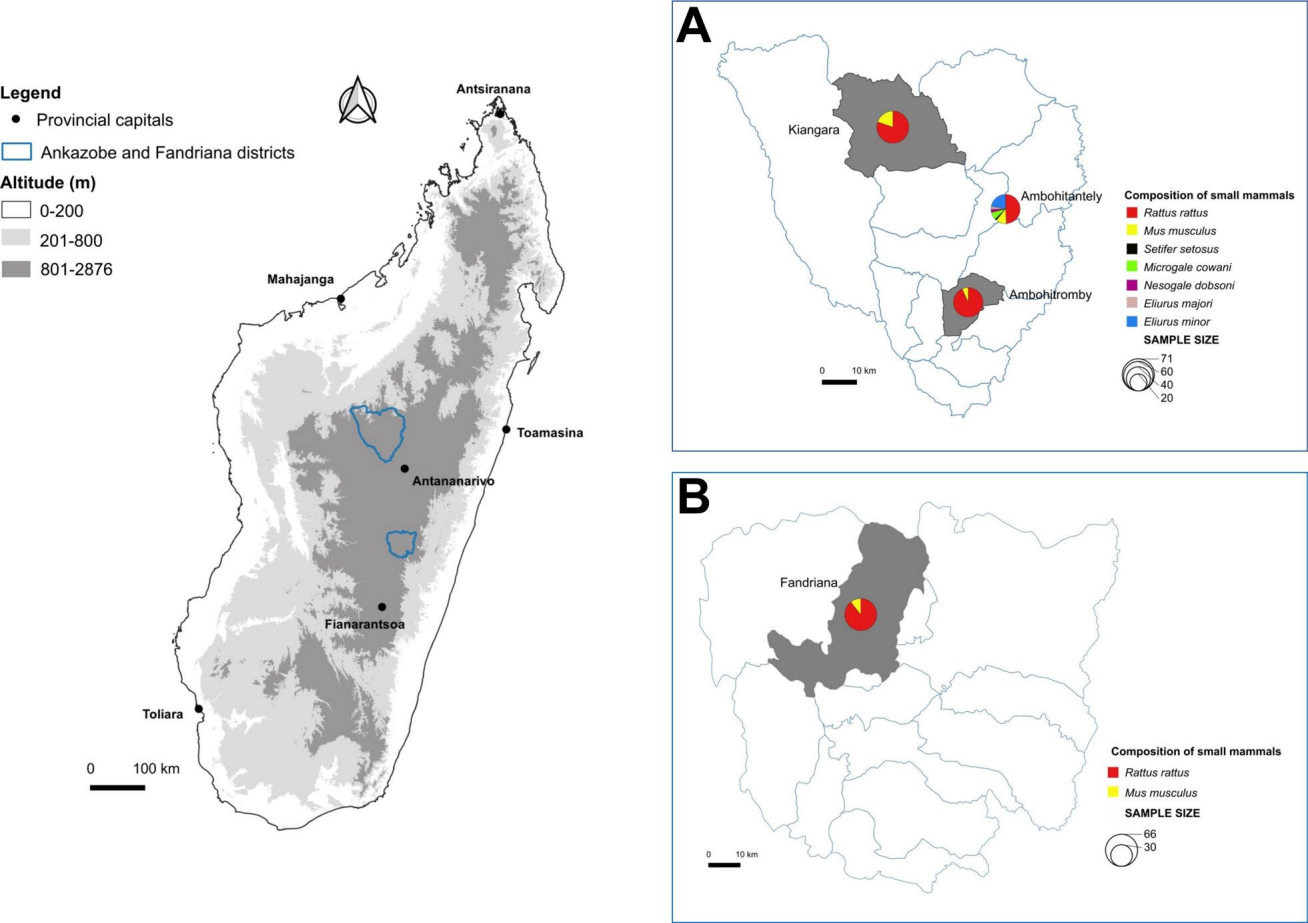
Map showing elevation gradients in (**A**) Ankazobe District and (**B**) Fandriana District in the Central Highlands of Madagascar

The study was carried out in two areas of Madagascar’s Central Highlands: Ankazobe District, located in the northern part of the former Antananarivo Province (now Analamanga Region), and Fandriana District, located in the northern part of former Fianarantsoa Province (now Amoron’i Mania Region). Small mammals were captured at four localities, representing two different habitats: rural zones with no natural forest cover (Ambohitromby at 18° 26.07′ S, 47° 10.12′ E, 1260 m elevation; Kiangara at 17° 58.31′ S, 47° 1.78′ E, 904 m elevation; and Fandanana at 20° 19.9′ S, 47° 25.9′ E, 1417 m elevation), and an area of natural montane forest (Réserve Spéciale d’Ambohitantely at 18° 10.26′ S, 47° 16.90′ E, 1595 m elevation) (Fig. 1).

### Small mammals and ectoparasite sampling

The study was conducted in strict accordance with the terms of a research permit issued by the Malagasy authority (Direction du Système des Aires Protégées, Direction Générale de l’Environnement et des Forêts) and associated field collecting permit (number 225/16/MEEF/SG/DGF/DSAP/SCB.Re, issued 19 September 2016).

Small mammals were captured during September and December 2016 using two types of traps: Sherman (H.B. Sherman Trap, Tallahassee, FL; 23 long × 7.5 width × 9 cm high) and National (Tomahawk Live Trap, Hazelhurst, WI; 39.2 long × 12.3 wide × 12.3 cm high). Each trap was baited with peanut butter, which was renewed daily. Small mammals were captured on three successive nights at each of the four sites. All captured animals were euthanized by cervical dislocation. Ectoparasites were removed by rigorously brushing the fur of each individual within a 30-cm-deep basin, and they were preserved in ethanol; the subsequent steps involved ectoparasite species identification and molecular screening. The ectoparasites species were identified morphologically using available taxonomic keys for fleas [21] and ticks [22].

### Biological data collection

Identification of small mammal species was performed using body measurements and available taxonomic keys [23]. Blood samples were collected by cardiac puncture, dropped onto filter paper disks (LDA22, Zoopole, Ploufragan, France), and centrifuged to obtain sera. A spleen sample for each individual were aseptically collected and conserved in Cary Blair medium.

For black rats (*Rattus rattus*), their age was inferred on the basis of different characters. For males, body weight, testicular position (abdominal or scrotal), and epididymes development (undeveloped or developed) were noted to estimate age class and reproductive status. For females, in addition to weight, mammae state (small, large or lactating), vaginal condition (perforated or imperforate), embryos (present or absent), and placental scars (absent or present) were used to extrapolate age class and reproductive status. Three age categories were used for black rats (juvenile, subadult, and adult) [24, 25].

### Molecular screening of* Bartonella*

The spleen sample was smashed with 1× phosphate-buffered saline (PBS). Genomic DNA was extracted according to the manufacturer’s protocol using QIAmp DNA mini kit (Qiagen, Germany). For ectoparasites, DNA extraction was performed using DNeasy Blood and Tissue kit (Qiagen, Germany) after mechanical grinding with the Tissue Lyser II (Qiagen, Germany). Subsequently, conventional PCR was carried out to amplify a 346-bp region of gamma subunit of nicotinamide adenine dinucleotide hydrogen (NADH) dehydrogenase (*nuoG*) of *Bartonella*, using a set of primers nuoG-F (5′-GCGTGATTGTTCTCGTTA-3′) and nuoG-R (5′-CACGACCACGGCTATCAAT-3′). Cycling conditions for the PCR included: 94 °C for 2 min, 45 cycles of 94 °C for 30 s, 55 °C for 1 min, 72 °C for 1 min, and a final extension of 72 °C for 7 min [26]. In addition to extraction control (no sample) during DNA extraction, positive and negative controls were also amplified to avoid the presence of false positive and false negative results. Amplicons obtained were run for electrophoresis on a 1.5% agarose gel under a 120-V generator for 90 min. PCR products were visualized using Gelscan (Vilber Lourmat, France) and subsequently sent to Genoscreen (Lille, France) for Sanger sequencing using the same primers as the initial PCR assay.

### Phylogenetic analysis

Nucleotide sequences obtained from *nuoG*-positive individuals were manually edited using Geneious Prime sequence alignment editor version 2024.0.7 [27]. The program Basic Local Alignment Search Tool (BLAST, https://blast.ncbi.nlm.nih.gov) [28] was used to compare identified nucleotide sequences with those available on GenBank. Before performing the phylogenetic analysis, the best substitution model was determined with jModelTest 2.1.10 [29] using the lowest Akaike information criterion score. Phylogenetic trees were constructed using maximum likelihood method, and bootstrap analyses were performed with 10,000 iterations using MEGA X software [30].

### Statistical analyses

Chi-squared tests were carried out to verify the difference in *Bartonella* infection according to host species and sex of small mammals. For *Rattus rattus*, we performed generalized linear models (GLM) using a binomial distribution to examine the infection risk of *Bartonella* according to the predictor variables (age class, sex, and locality). Selection of the best model was based on the Akaike information criterion (AIC). Odds ratios and *p*-values indicate correlations of *Bartonella* infection in *R. rattus* with the analyzed variables. Statistical analyses were performed using R software [31].

## Results

### Molecular detection of *Bartonella* in small mammals

A total of 253 spleen-derived DNA samples from small mammals were tested by conventional PCR targeting the *nuoG* gene of *Bartonella*. Of these animals, 152 individuals were infected, with an overall prevalence of 60.1% (152/253), with 54.1% (137/253) of these infected individuals being *Rattus rattus*. Overall infection rates varied between 48.3% and 80.9% at the four study sites, ranging from 48.3% (29/60) at Ambohitromby, 52.1% (37/71) at Kiangara, 59.3% (35/59) at Ambohitantely, to 80.9% (51/63) at Fandriana (Table 1). According to host species, 92.3% (12/13) of *Eliurus minor*, 69.1% (137/198) of *R. rattus*, 50.0% (1/2) of *E. majori*, 50.0% (1/2) of *Nesogale dobsoni*, and 20.0% (1/5) of *Microgale cowani* were infected by *Bartonella* (Table 1). Variation in *Bartonella* prevalence was significant associated with host species (chi-squared test, *χ*^2^ = 65.67, *df* = 6, *P* < 0.001, Table 1). In analyses of infected individuals at the four localities based on sex, 62.4% (78/125) of individual females and 57.8% (74/128) of individual males were not statistically significant (chi-squared test, *χ2* = 0.554, *df* = 1, *P* = 0.456, Table 1)*.*

**Table 1:** Prevalence of *Bartonella* in small mammals collected in the Central Highland areas of Madagascar

Parameter		Location				Total (%)	*df*	*χ*^2^ value	*P*
		Forest	Rural						
		Ambohitantely	Ambohitromby	Kiangara	Fandriana				
Family	Species						6	65.67	0.00001
Muridae	*Mus musculus*	0/7	0/4	0/14	0/7	**0/32**			
	*Rattus rattus*	20/29 (68.9)	29/56 (51.7)	37/57 (64.9)	51/56 (91)	**137/198 (69.1)**			
Nesomyidae	*Eliurus majori*	1/2 (50.0)	–	–	–	**1/2 (50.0)**			
	*E. minor*	12/13 (92.3)	–	–	–	**12/13 (92.3)**			
Tenrecidae	*Microgale cowani*	1/5 (20.0)	–	–	–	**1/5 (20.0)**			
	*Nesogale dobsoni*	1/2 (50.0)	–	–	–	**1/2 (50.0)**			
	*Setifer setosus*	0/1	–	–	–	**0/1**			
	Sex						1	0.55	0.456
	Female	17/26 (65.3)	12/29 (41.3)	22/36 (61.1)	27/34 (79.4)	**78/125 (62.4)**			
	Male	18/33 (54.5)	17/31 (54.8)	15/35 (42.8)	24/29 (82.7)	**74/128 (57.8)**			
	Overall prevalence	**35/59 (59.3)**	**29/60 (48.3)**	**37/71 (52.1)**	**51/63 (80.9)**	**152/253 (60.1)**			

DNA-positive specimens of *Bartonella* / total specimens (prevalence %); “–” indicates that no animal of this species was captured

**Fig. 2:**
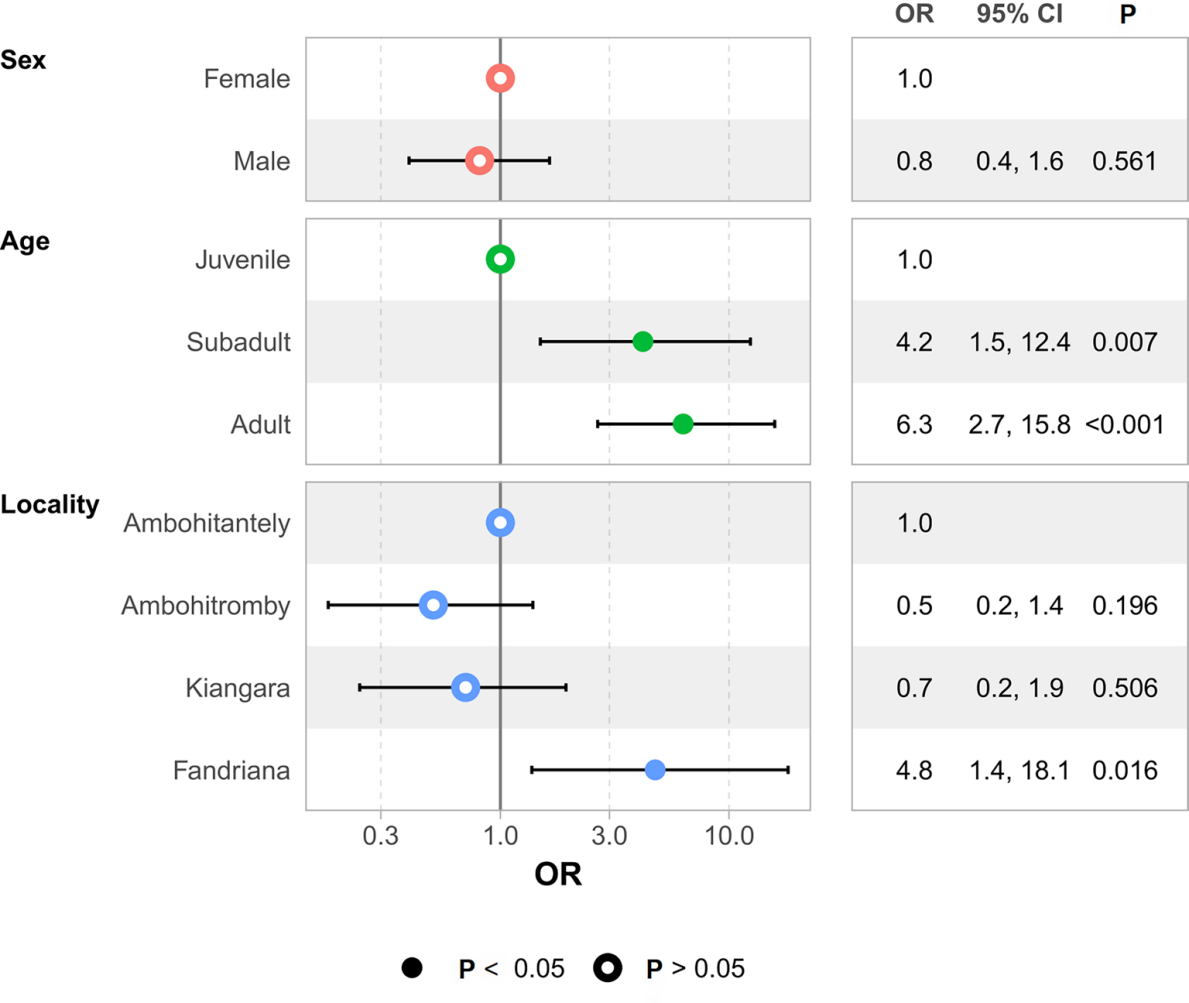
Factors associated with *Bartonella* infection in *Rattus rattus* by sex, age class, and different study sites in the Central Highlands of Madagascar. The odds ratio (OR) measure is the chance of *Bartonella* infection relative to a reference group

For *R. rattus*, 51.7% (29/56) were infected by *Bartonella* at Ambohitromby, 64.9% (37/57) at Kiangara, 68.9% (20/29) at Ambohitantely, and 91.0% (51/56) at Fandriana. Using GLM analysis, we found significant differences in *R. rattus* of *Bartonella* infection rates at the sampled localities (*P* = 0.016) (Fig. 2). According to *R. rattus* age classes, 36.1% (13/36) of juveniles, 78.4% (40/51) of subadults, and 75.6% (84/111) of adults were positive (*P* < 0.001) (Fig. 2). Further, on the basis of sex, 71.7% (71/99) of individual females and 66.6% (66/99) of individual males were infected by *Bartonella* (*P* = 0.56) (Fig. 2).

### Molecular detection of *Bartonella* in ectoparasites of small mammals

For ectoparasites, 132 individual fleas comprising five species (three introduced *Xenopsylla cheopis*, *Pulex irritans*, and *Echidnophaga gallinacea*, and two endemic *Synopsyllus fonquerniei* and *Dinopsyllus brachypecten*) were tested by conventional PCR. Among the 132 DNA flea samples, 15.9% (21/132) tested positive with overall prevalence of 17.5% (7/40) at Fandriana, 17.4% (11/63) at Kiangara, 11.1% (1/9) at Ambohitantely, and 10.0% (2/20) at Ambohitromby. Fleas were collected from *R. rattus* at all four localities. According to flea species, infection rates of *Bartonella* were 21.2% (14/66) for *S. fonquerniei*, 15.7% (3/19) for *X. cheopis*, 11.7% (2/17) for *D. brachypecten*, 3.4% (1/29) for *E. gallinacea*, and 100% (1/1) for *P. irritans*. When found on small mammal species, *S. fonquerniei* were infected at all four localities (Table 2). For ticks, 51 individuals composed of seven species (*Haemaphysalis* sp., *H. elongata*, *Ixodes* sp. 1, *Ixodes* sp. 2, *I. lunatus*, *I. nesomys*, and *I. colasbelcouri*) were tested by conventional PCR. *Bartonella* was not detected in any of the tested tick samples (Table 2).

**Table 2:** Prevalence of *Bartonella* in ectoparasite of small mammals collected in the Central Highlands of Madagascar (*N*: number of individuals, Prev: prevalence)

	Host	Ambohitantely						Ambohitromby		Kiangara		Fandriana	
		*Rattus rattus*		*Eliurus minor*		*Setifer setosus*		*R. rattus*		*R. rattus*		*R. rattus*	
		*N*	Prev	*N*	Prev	*N*	Prev	*N*	Prev	*N*	Prev	*N*	Prev
Fleas	*Synopsyllus fonquerniei*	7	1/7 (14.2)	1	0/1	1	0/1	12	2/12 (16.6)	23	7/23 (30.4)	22	4/22 (18.1)
	*Xenopsylla cheopis*	–	–	–	–	–	–	4	0	15	3/15 (20.0)	–	–
	*Echidnophaga gallinacea*	–	–	–	–	–	–	4	0	25	1/25(4.0)	–	–
	*Pulex irritans*	–	–	–	–	–	–	–	–	–	–	1	1/1
	*Dinopsyllus brachypecten*	–	–	–	–	–	–	–	–	–	–	17	2/17(11.7)
Ticks	*Haemaphysalis* sp.	–	–	–	–	–	–	–	–	–	–	22	0/22
	*H. elongata*	–	–	–	–	1	0/1	–	–	–	–	–	–
	*Ixodes* sp. 1	–	–	–	–	–	–	–	–	–	–	12	0/12
	*Ixodes* sp. 2	–	–	–	–	–	–	–	–	–	–	1	0/1
	*I. lunatus*	–	–	–	–	–	–	–	–	–	–	1	0/1
	*I. nesomys*	–	–	–	–	–	–	–	–	–	–	2	0/2
	*I. colasbelcouri*	12	0/12	–	–	–	–	–	–	–	–	–	–

DNA-positive specimens of *Bartonella* / total specimens (prevalence %); “–” indicates that no ectoparasite of this species was collected

### BLAST results and phylogenetic analysis of *Bartonella* species

A total of 22 *Bartonella nuoG* sequences were obtained and compared with those deposited in GenBank to assess sequence similarity. Among them, eight sequences, viz. two from *Rattus rattus*, five from *Synopsyllus fonquerniei*, and one from *Dinopsyllus brachypecten* showed 100% similarity with sequences of uncultured *Bartonella* sp. deposited in GenBank (JX131676). Two sequences, one from *Echidnophaga gallinacea* and one from *S. fonquerniei*, also showed 100% similarity with *B. kosoyi* (CP031843). Four sequences from *Eliurus minor* showed 95% similarity with *Bartonella* sp. (OR187492). Seven sequences, viz. five from *R. rattus*, one from *S. fonquerniei*, and one from *Xenopsylla cheopis*, highlighted 100% similarity with uncultured *Bartonella* sp. (MF498850). One sequence from *Nesogale dobsoni* showed 89% similarity with *Bartonella* sp. (MN529408).

**Fig. 3:**
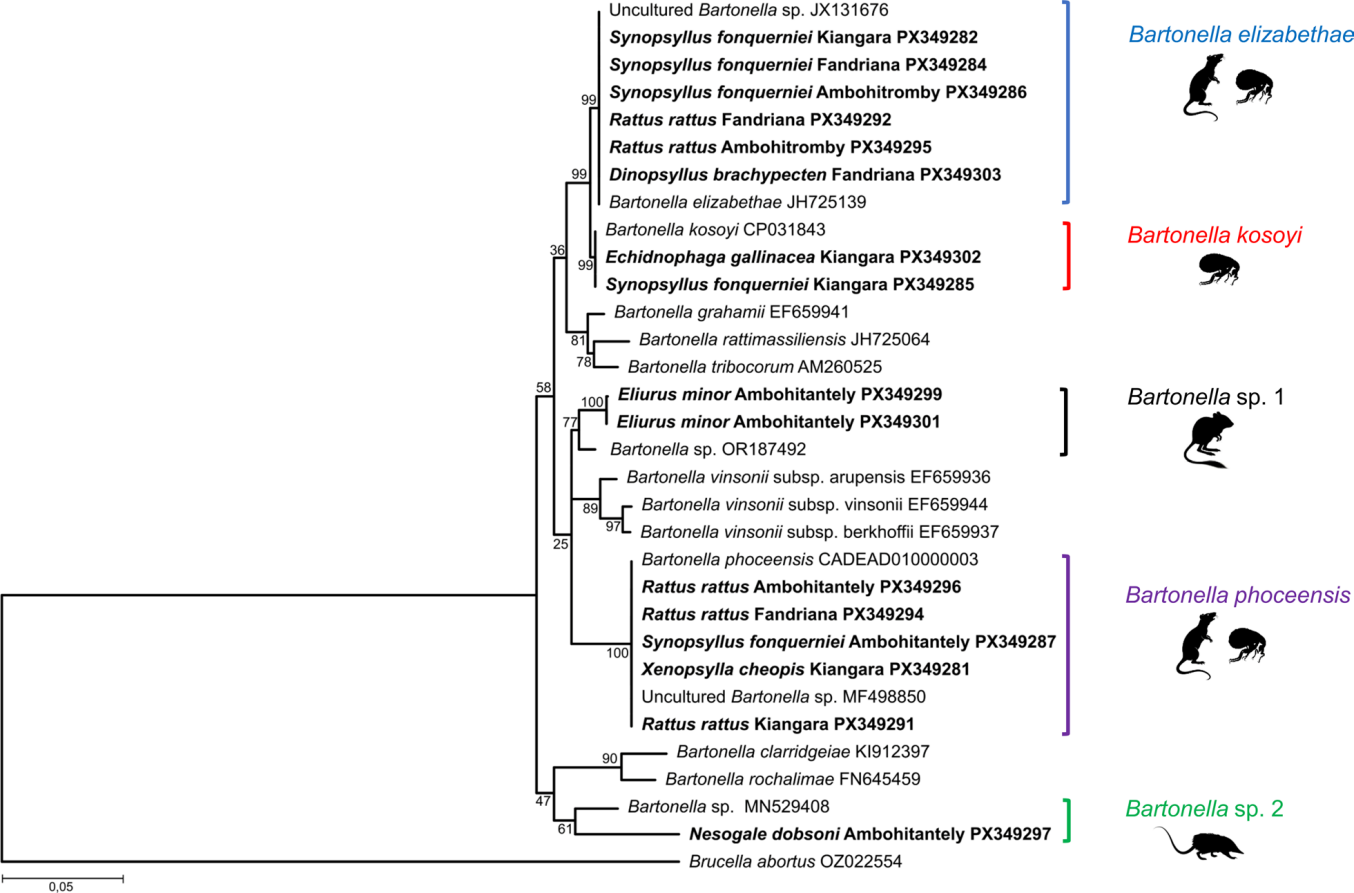
Phylogenetic reconstruction of *Bartonella* species in small mammals and fleas collected in Ankazobe and Fandriana district using maximum likelihood method on the basis of the *nuoG* gene. The phylogenetic tree was performed using Tamura–Nei model (TrN + I + G) as the best substitution model according to jModelTest with 10,000 iterations. Scale bar indicates the number of substitutions per site. Sequences obtained in this study are highlighted in bold

On the basis of the phylogenetic analysis (maximum likelihood method and Tamura–Nei model), five clades of *Bartonella* were identified. The first falls within *B. elizabethae* (JH725139) identified in *R. rattus*, *D. brachypecten*, and *S. fonquerniei* from Ambohitromby, Fandriana, and Kiangara. The second clade, which is referable to *B. kosoyi* (CP031843), was identified in *S. fonquerniei* and *E. gallinacea* from Kiangara. The third clade included a *Bartonella* sequence from *E. minor* collected in Ambohitantely, which clustered near *Bartonella* sp. (OR187492). The fourth clade corresponded to *B. phoceensis* (CADEAD010000003) and included *R. rattus* and two species of fleas, *S. fonquerniei* from Ambohitantely and *X. cheopis* from Kiangara. The last clade, with a *Bartonella* sequence from *N. dobsoni* collected in Ambohitantely, grouped with *Bartonella* sp. (MN529408). New sequences obtained in this study were deposited in GenBank under accession numbers PX349281 to PX349303 (Fig. 3, Additional file 1).

## Discussion

Small mammals contribute to ecosystem functioning on the basis of a number of natural history aspects, including seed dispersal, regulating arthropod populations, playing a role in the food chain (as invertebrate predators and carnivore animal prey), and in the maintenance and transmission of zoonotic agents within their community and to humans [32]. Madagascar is home to 64 species of nonvolant small mammals, grouped in three orders (Afroscoricida, Soricomorpha, and Rodentia), with an endemism rate of 92% [33]. Invasive and introduced rodents such as *Rattus rattus* are the most abundant species in rural and forest habitats, possibly being responsible for the decline of endemic rodent species through competition or disease transmission [34]. Moreover, *R. rattus* host numerous zoonotic agents such as *Yersinia pestis*, which have a notable impact on public health in Madagascar, particularly in the Central Highlands [35].

As reported herein, the presence of *Bartonella* spp. was confirmed on the basis of molecular tools in endemic rodents (Nesomyidae: *Eliurus majori* and *E. minor*), endemic tenrecs (Tenrecidae: *Microgale cowani* and *Nesogale dobsoni*), introduced rodents (Muridae: *R. rattus*), and their associated fleas (Pulicidae: *Synopsyllus fonquerniei*, *Echidnophaga gallinacea*, *Pulex irritans*, and *Xenopsylla cheopis*, and Ctenophthalmidae: *Dinopsyllus brachypecten*). According to age class, our data indicate notable variation of *Bartonella* infection rates in *R. rattus*, with subadults and adults having rates than juveniles. Krügel et al. [36] also found this same relationship in *Bartonella* infection rates of *R. rattus*, but with a two-category age classification, with juveniles being significantly less infected than adults. The life cycle of *Bartonella* species, such as *B. tribocorum*, can explain why older animals are more likely to be infected by *Bartonella*, as this bacterium can be maintained in the host without apparent deleterious consequences [37, 38]. Regarding differences between sampling localities, our study showed a higher prevalence of *Bartonella* at Fandriana, which may be explained by the greater diversity and abundance of ectoparasites observed in captured *R. rattus* at this locality (Table 2).

The density of vectors is a major factor influencing *Bartonella* prevalence and transmission within small mammal communities or in humans [4]. Furthermore, all flea species tested in this study were infected by *Bartonella*, but with different prevalence rates. The endemic flea, *S. fonquerniei*, is the most frequently infected by *Bartonella* at the four study localities. A previous study undertaken at Ambohitantely also showed higher *Bartonella* infection rates in *S. fonquerniei*, as compared with other rodent flea species [19], but the vectorial competence of this endemic flea in the transmission of *Bartonella* remains unknown.

On the basis of our phylogenetic inference, three species of *Bartonella* were identified (*B. kosoyi*, *B. elizabethae*, and *B. phoceensis*), as well as two unidentified clades from endemic mammal species (*Eliurus minor* and *Nesogale dobsoni*). To the best of our knowledge, this is the first detection of *B. kosoyi* in Madagascar. These three identified species of *Bartonella* were generally hosted by *R. rattus*, with *B. kosoyi* and *B. elizabethae* recognized as potential zoonotic agents [8, 39]. Furthermore, our study along with that conducted by Brook et al. [19] confirmed the presence of four potentially zoonotic *Bartonella* species (*B. tribocorum*, *B. rattimassiliensis*, *B. elizabethae*, and *B. kosoyi*) identified in *R. rattus* and their ectoparasites in Madagascar. Although no human cases of bartonellosis have been reported on the island, these pathogens constitute a potential zoonotic threat associated with anthropogenic pressure and climatic change. Ecological degradation increases the risk of contact between humans, reservoirs, and vectors, and facilitates the maintenance and transmission of vector-borne diseases such as bartonellosis. However, on Madagascar, our knowledge is limited of *Bartonella* epidemiology, its distribution, as well as genetic diversity of rodent-borne zoonotic diseases. Metagenomic analysis of rodent-associated pathogens for countries such as Madagascar are needed to elucidate the presence, abundance, and areas at risk of rodent-borne diseases. These different aspects taken together, and how little is known about bartonellosis risks in Madagascar, highlight the need for further study on the diversity and drivers of infection related to rodent-related zoonotic disease surveillance on the island and identify their potential impact on public health.

## Conclusions

This study extends our knowledge on the prevalence and diversity of *Bartonella* species in small mammal and their ectoparasites in the Central Highlands of Madagascar. Molecular analyses confirmed the presence of *Bartonella* in four endemic small mammal species (*Eliurus minor*, *E*. *majori*, *Microgale cowani*, and *Nesogale dobsoni*), in one introduced species (*Rattus rattus*), and their associated fleas (*Synopsyllus fonquerniei*, *Dinopsyllus brachypecten*, *Echidnophaga gallinacea*, *Pulex irritans*, and *Xenopsylla cheopis*) with high prevalence in *Eliurus minor*, *R. rattus*, and *S. fonquerniei*. Two potential zoonotic agents (*B. kosoyi* and *B. elizabethae*) were identified in *R. rattus* and their fleas, highlighting their potential public health importance. Future work incorporating additional molecular markers and broader geographic sampling are essential to better assess the diversity of *Bartonella* species on the island and to achieve a more comprehensive understanding of their spatial distribution. Research should also evaluate the impact of environmental and anthropogenic factors on the maintenance and transmission of the bacterial genus through a multidisciplinary “One Health” approach and assess their potential impacts on public health.

## Supplementary Information

**Additional file 1.**

## Data Availability

All data analyzed during this study are included in this published article and its supplementary information files.
